# Endovascular versus surgical treatment of cranial dural arteriovenous fistulas: a single-center 8-year experience

**DOI:** 10.1007/s00701-021-04950-9

**Published:** 2021-09-06

**Authors:** Wilhelm Sorteberg, Angelika Sorteberg, Eva Astrid Jacobsen, Pål Rønning, Terje Nome, Per Kristian Eide

**Affiliations:** 1 Department of Neurosurgery, Oslo University Hospital – Rikshospitalet, P.B. 0454 Nydalen, 0424 Oslo, Norway; 2 Institute of Clinical Medicine, Faculty of Medicine, University of Oslo, Oslo, Norway; 3 Department of Radiology and Nuclear Medicine, Oslo University Hospital – Rikshospitalet, Oslo, Norway

**Keywords:** Dural arteriovenous fistula, Intracranial hemorrhage, Cerebral venous aneurysm, Treatment, Outcome

## Abstract

**Background:**

Cranial dural arteriovenous fistulas (dAVFs) are rare lesions managed mainly with endovascular treatment (EVT) and/or surgery. We hypothesize that there may be subtypes of dAVFs responding better to a specific treatment modality in terms of successful obliteration and cessation of symptoms and/or risks.

**Methods:**

All dAVFs treated during 2011–2018 at our hospital were analyzed retrospectively. Presenting symptoms, radiological variables, treatment modality, complications, and residual symptoms were related to dAVF type using the original Djindjian classification.

**Results:**

We treated 112 dAVFs in 107 patients (71, 66% males). They presented with hemorrhage (*n* = 23; 21%), non-hemorrhagic symptoms (*n* = 75; 70%), or were discovered incidentally (*n* = 9; 8%). There were 25 (22%) type I, 29 (26%) type II, 26 (23%) type III, and 32 (29%) type IV fistulas. EVT was the primary treatment modality in 72/112 (64%) dAVFs whereas 40/112 (36%) underwent primary surgery with angiographic obliteration rates of 60% and 90%, respectively. Using a secondary treatment modality in 23 dAVFs, we obtained a final obliteration rate of 93%, including all type III/IV and 26/27 (96%) type II dAVFs. Except for headache, residual symptoms were rare and minor. Permanent neurological complications consisted of five cranial nerve deficits.

**Conclusions:**

We recommend EVT as first treatment modality in types I, II, and in non-hemorrhagic type III/IV dAVFs. We recommend surgery as first treatment choice in acute hemorrhagic dAVFs and as secondary choice in type III/IV dAVFs not successfully occluded by EVT. Combining the two modalities provides obliteration in 9/10 dAVF cases at a low procedural risk.

## Introduction

A cranial dural arteriovenous fistula (dAVFs) is a rare type of neurovascular lesion with a detection rate of 0.16/100,000 adult years [1]. They may be discovered incidentally, but depending on their location and venous drainage, they may cause pulsatile tinnitus, headache, ocular and neurological symptoms, or intracranial hemorrhage [8, 12, 19, 20, 22, 23, 26, 29]. Once discovered, dAVFs are delineated using cerebral six-vessel digital subtraction angiography (DSA) and categorized according to their drainage into dural sinuses and leptomeningeal veins. The most common classifications are the Djindjian, Borden, Cognard, and the Directness, Exclusivity, and Strain (DES) [3–5, 7, 10, 13].

The choice of treatment is based on presenting symptoms and the dAVF angioarchitecture. Although endovascular treatment (EVT) has become the primary treatment modality of most cranial dAVFs [2, 19, 20, 26, 29], surgery is used in various settings [5, 20, 31, 32]. The advantage of each treatment modality in relation to the type of dAVF and clinical setting is, however, not fully delineated. By presenting our experience of dAVF treatment with both treatment modalities, we want to expand the existing knowledge by systemising their use in accordance with dAVF type and clinical setting. The hypothesis is that there may be subtypes of dAVF responding better to a specific treatment modality in terms of successful obliteration and cessation of symptoms and/or risks. To this end, we retrospectively analyzed the clinical, radiological, treatment, and complication data of all patients with cranial dAVFs treated with EVT and/or surgery at our hospital during the 8-year period 2011–2018.

## Material and methods

### Infrastructure

Oslo University Hospital has been designated to be the national referral center for EVT in neurovascular malformations by the Norwegian Health Authorities. The vast majority of cranial dAVFs in Norway (population 5.3 million 2020; www.ssb.no) are therefore treated at our institution.

Our goals for treatment are prevention of intracranial hemorrhage/rebleed, reversal of neurological/ocular symptoms, and palliation of intolerable symptoms due to the dAVF blood flow.

Elective cases are evaluated in weekly meetings by our neurovascular team consisting of neuro-interventionists from the Department of Radiology and vascular neurosurgeons from the Department of Neurosurgery. For each patient, detailed medical history and the results of clinical neurological examination are considered together with the radiological findings. Emergencies are managed by the vascular neurosurgeon and neuro-interventionist on call.

### Patients and data collection

All adult patients with cranial dAVFs in whom endovascular/surgical treatment was initiated and completed at our hospital from January 2011 through December 2018 were included in this retrospective study. Excluded were eight patients that had treatment of Barrow type A high-flow carotid-cavernous fistulas [6], two patients that received part of their treatment at other hospitals, and one patient in whom treatment was not completed until 2019.

Data were retrieved from medical records, PACS (Sectra® AB, Linköping, Sweden), and surgical protocols. We registered demographic data, clinical presentation, dAVF characteristics (lesion location and venous drainage), and treatment modality. Treatment was evaluated according to residual symptoms, angiographic dAVF obliteration, and procedural complications.

The study was approved as a quality control project by the institutional data protection officer (number 2012/14909) and exempt from informed consent.

### dAVF classification

**Fig. 1:**
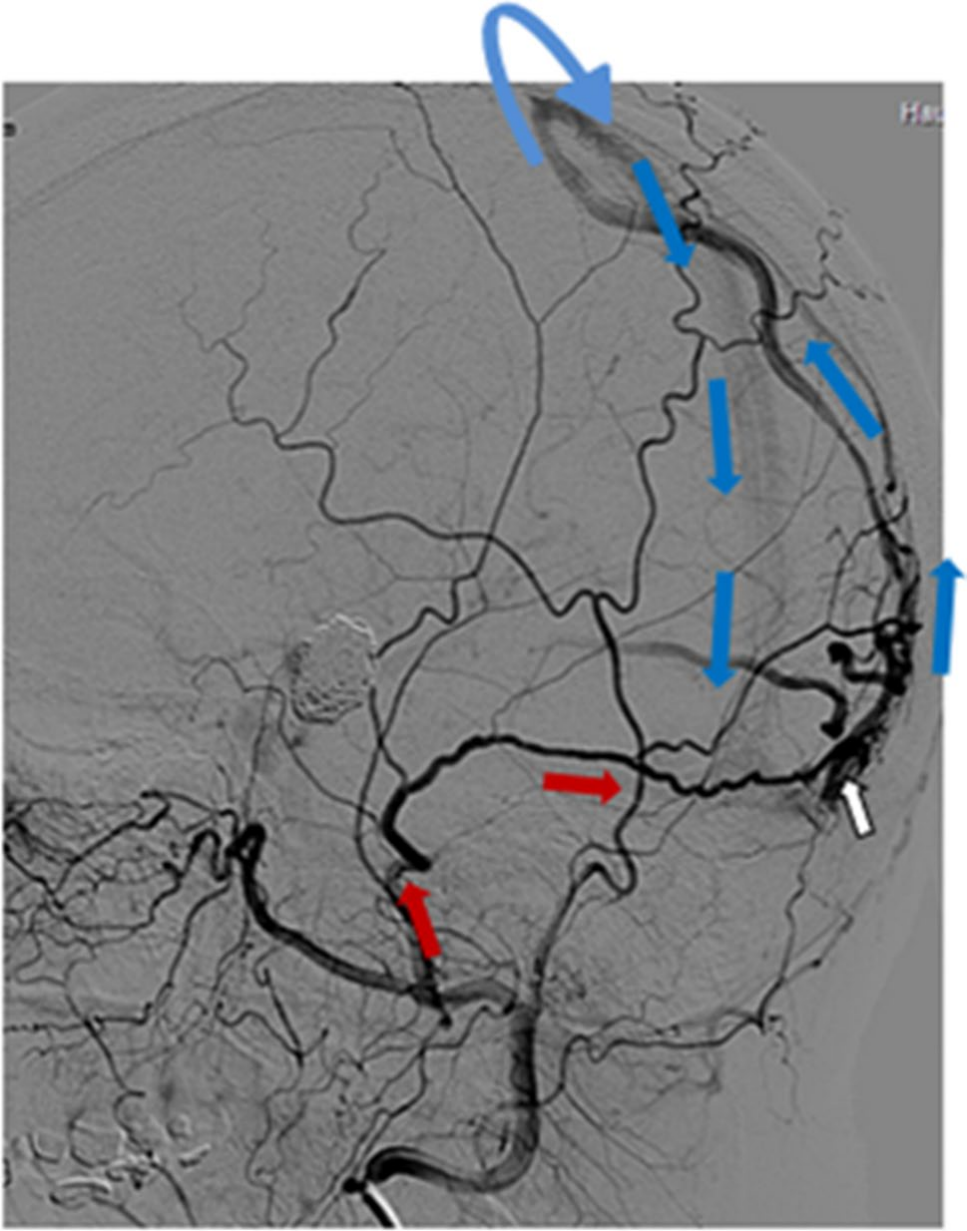
Oblique projection of a dAVF (white arrow) with leptomeningeal drainage of Djindjian type III. Cerebral angiography with contrast in the occipital artery supplying the fistula (red arrows) and retrograde venous drainage to the superior sagittal sinus (blue arrows)

**Fig. 2:**
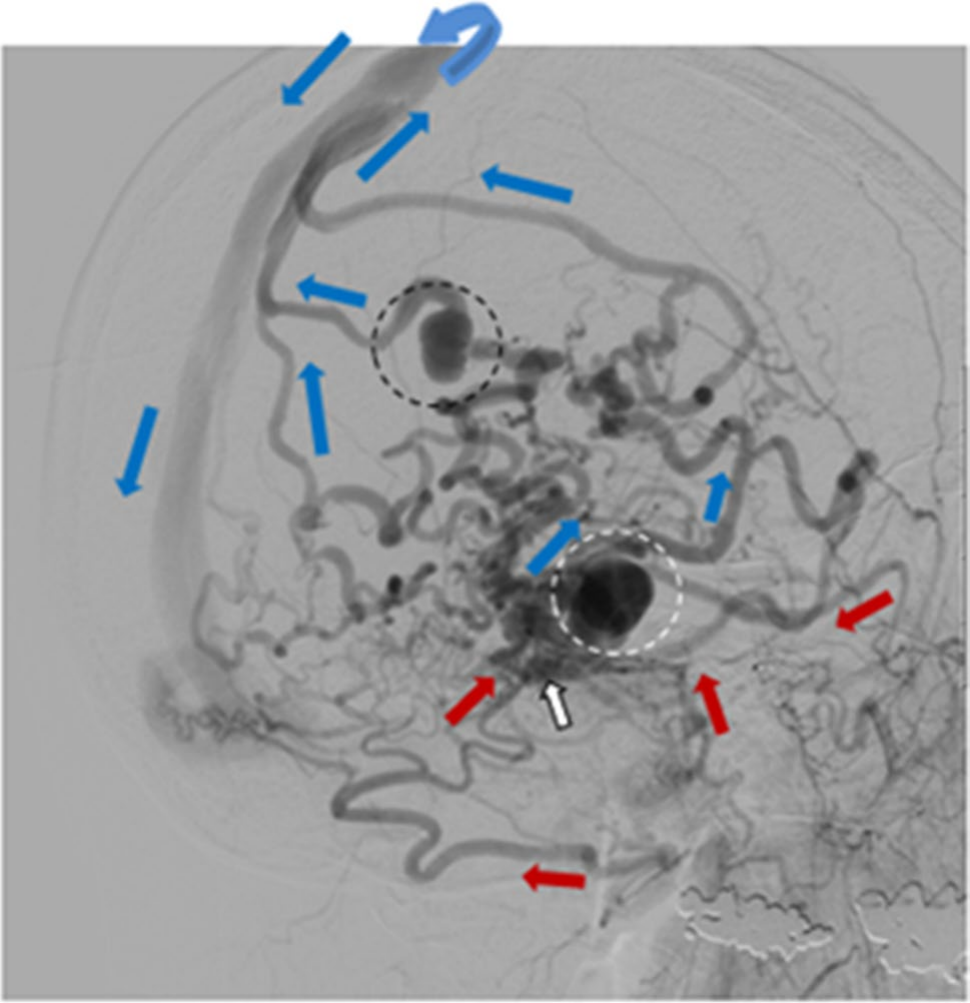
Oblique projection of a dAVF (white arrow) with leptomeningeal drainage of Djindjian type IV. Cerebral angiography with contrast in the occipital artery (left two red arrows) and middle meningeal artery (right two red arrows) supplying the fistula and retrograde venous drainage to the superior sagittal sinus (blue arrows) with venous ectasias/aneurysms (circles) on the draining veins

We categorized the dAVFs according to the original classification system that Djindjian et al. introduced in 1977 [13], which is the system Borden et al. based their classification of 1995 on [7]. The subtypes are presented in Table 1, also including the Cognard classification [10]. While types I and II correspond in the Djindjian and Borden systems, Borden type III dAVF draining into leptomeningeal vein(s) was in the Djindjian classification differentiated into types III and IV, depending on the absence or presence of aneurysmatic/ectatic segment(s) on the leptomeningeal draining vein(s) (Table 1). An example of Djindjian type III (corresponding to Borden type 3) is shown in Fig. 1, and an example of Djindjian type IV (corresponding to Borden type 3) is shown in Fig. 2.

**Table 1:** Current classifications of dAVFs

Type	Djindjian [13]	Borden [7]	Cognard [10]
I	Normal antegrade drainage into dural sinus or meningeal veins only	Normal antegrade drainage into dural sinus or meningeal veins only	Normal antegrade drainage into dural sinus or meningeal veins only
II	Drainage both into dural sinus and retrograde into leptomeningeal vein(s)	Drainage both into dural sinus and retrograde into leptomeningeal vein(s)	a) Retrograde drainage into dural sinus(es) b) Retrograde drainage into cortical vein(s) a + b) Retrograde drainage into dural sinus(es) and cortical vein(s)
III	Exclusive retrograde drainage into leptomeningeal vein(s) without any aneurysmatic or ectatic venous segments	Exclusive retrograde drainage into leptomeningeal vein(s) No differentiation whether aneurysmatic or ectatic venous segments are present or not	Exclusive retrograde drainage into leptomeningeal vein(s) without venous ectasia
IV	Exclusive retrograde drainage into leptomeningeal vein(s) with aneurysmatic or ectatic venous segments		Exclusive retrograde into leptomeningeal vein(s) with venous ectasia > 5 mm and × 3 larger than diameter of draining vein
V			Drainage to spinal perimedullary veins

**Fig. 3:**
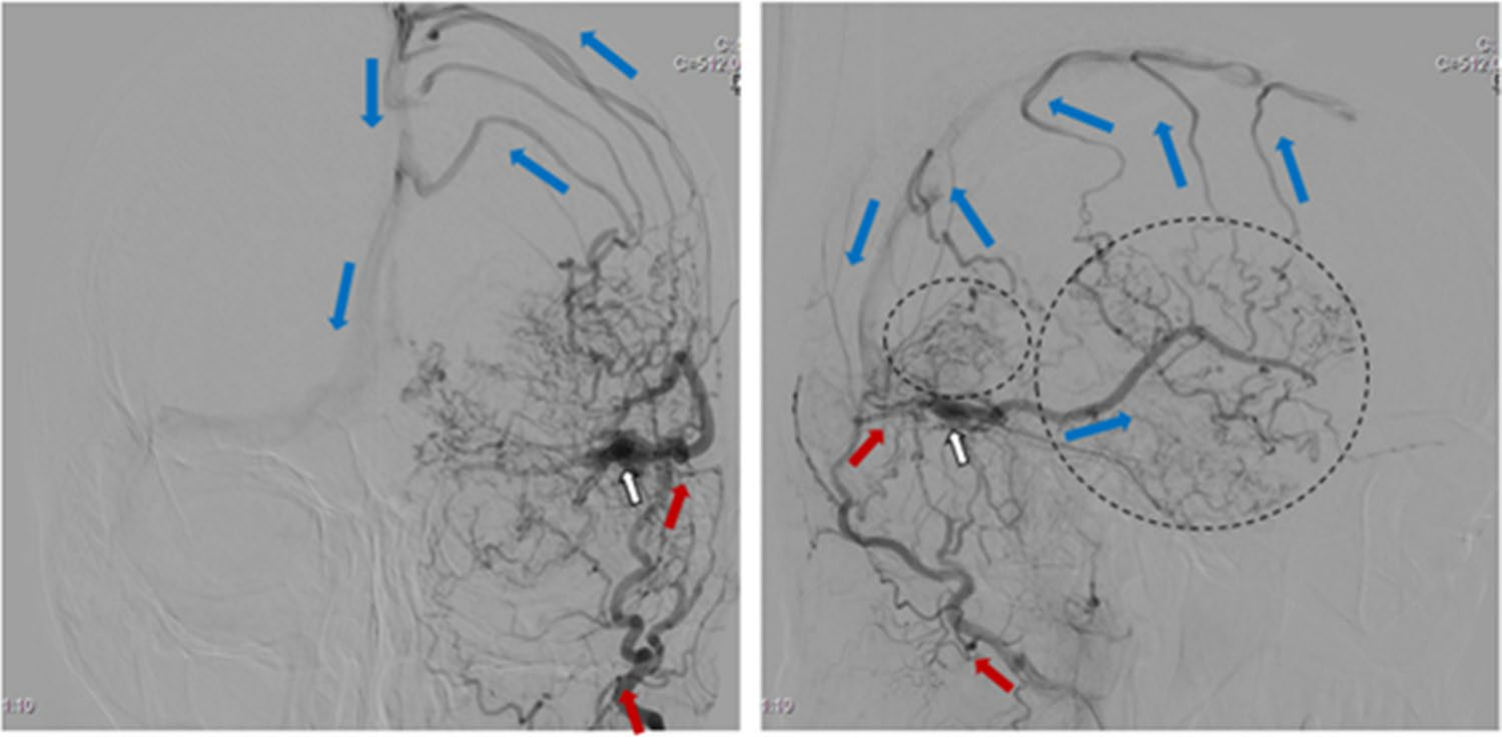
Cerebral angiography (anterior–posterior and lateral projections) showing a dAVF (white arrow) with pseudophlebitic venous appearance (circles) of leptomeningeal drainage (blue arrows). Arterial supply from the occipital artery (red arrows)

Hemorrhagic cases were also evaluated with regard to the presence of a congestive pseudophlebitic venous appearance [3] (see Fig. 3).

### Institutional management principles

In general, new patients undergo a (new) preoperative six-vessel DSA at our institution to delineate the dAVF prior to treatment. If the clinical state in acute cases requires emergency hematoma evacuation, this is performed immediately after arrival, based on cerebral CT/CTA without a preoperative DSA.

Embolization of a dAVF aims to occlude the fistula by access through arterial feeders and/or a draining vein by occluding the foot of the draining vein(s) [19]. Selection of catheters and embolic materials depends on access route(s) and the dAVF angioarchitecture. Usually, a dimethyl sulfoxide (DMSO)-compatible micro catheter is positioned close to the fistulous site and DMSO-compatible embolic material is injected with or without balloon protection of the sinus, and with or without additional occlusion of draining venous ectasias with coils. We usually prefer the transvenous route for cavernous sinus dAVFs due to the combination of a higher clinical and anatomic cure and a lower incidence of complications compared to the transarterial route [29].

The preferred surgical treatment of type III/IV dAVFs is disconnection of the leptomeningeal vein(s) where it (they) emerge(s) from the fistula, thereby ensuring all routes of venous outflow are obliterated [11, 15]. In type II dAVFs, one may perform dAVF excision with resection of the sinus and not just venous disconnection. In patients with distal tortuous arterial feeders to dAVFs that cannot be reached adequately through transarterial endovascular routes, one may in rare cases perform surgical palliative distal extracranial feeding artery disconnection.

Patients that underwent elective embolization were routinely discharged to home the day after treatment. Most patients with some residual dAVF flow at the end of the EVT underwent control DSA at 3 months. After elective surgery, patients routinely underwent cerebral CT/CTA on the first postoperative day and postoperative DSA during the primary stay or at a follow-up visit. Our management of patients with acute intracranial hemorrhage from cranial dAVFs has been described recently [31].

### Statistics

Statistical analysis was performed using SPSS version 25 (IBM Corporation, Armonk, NY, USA). Not normal distributed continuous variables are presented with median and range Categorical variables are presented as frequencies or percentages, and the Chi square test was used to compare differences between independent groups, whereas one-sample binomial tests were used to assess differences in frequencies within one category. Two tailed *p*-values of less than 5% were considered statistically significant.

## Results

### Patients

Table 2 shows demographic information, clinical presentation, dAVF type, treatment modality, and management results. The median time from start of symptoms/incidental finding to treatment was 7 months (range 0 days–48 months).

**Table 2:** Patients, presentation, dAVF type, treatment, and results versus treatment modality in 107 patients with 112 dAVFs

		Treatment modality		*P*-value
	Total	Primary EVT	Primary surgery	
Demographic information				
Number	107	67	40	0.012
Gender male%/female%	66%/33%	52%/48%	90%/10%	< 0.001
Age (years)	56.1 ± 13.1	54.4 ± 14.3	59.1 ± 10.1	0.072
Clinical presentation				
Hemorrhage	23	6 (26%)	17 (74%)	0.035
Non-hemorrhagic symptoms	75	56 (75%)	19 (25%)	< 0.001
Incidental	9	5 (56%)	4 (44%)	1.000
dAVFs				
Type I	25	24 (96%)	1 (4%)	< 0.001
Type II	29	25 (86%)	4 (14%)	< 0.001
Type III	26	13 (50%)	13 (50%)	1.000
Type IV	32	10 (31%)	22 (69%)	0.052
Treatment modality				
Primary treatment	112	72 (64%) (primary EVT)	40 (36%) (primary surgery)	0.003
Secondary treatment	23	20 (87%) (secondary surgery)	3 (13%) (secondary EVT)	< 0.001
Management results				
Primary treatment occlusion	79/112 (71%)	43/72 (60%)	36/40 (90%)	0.001
Secondary treatment occlusion	19/23 (83%)	16/20 (75%)	3/3 (100%)	0.394
Late spontaneous occlusion after primary/secondary treatment	6	5	1	1.000
Final occlusion	104/112 (93%)	64/72 (89%)	40/40 (100%)	0.029

EVT was predominantly used as primary treatment in type I and type II dAVFs, whereas there was no predominance in type III and a trend to surgery in type IV dAVFs. Surgery was chosen as primary treatment in most hemorrhagic cases.

Predisposing factors for dAVF were unknown in 90/107 (84%) patients, whereas seven (7%) had a history of facial/head injury, five (5%) of dural sinus thrombosis, three (3%) of cranial neurosurgery, and two (2%) of central nervous system infection.

### Clinical presentation versus dAVF type

All hemorrhagic dAVFs drained into leptomeningeal veins only (type III (*n* = 2) and types IV (*n* = 21)), whereas 2/9 incidentally discovered fistulas drained also into a venous sinus (type II (*n* = 2), type III (*n* = 3), and type IV (*n* = 4)). Type I dAVFs were merely found in non-hemorrhagic symptomatic patients; these 75 patients harbored 80 fistulas: type I (*n* = 25), type II (*n* = 27), type III (*n* = 21), and type IV (*n* = 7).

### dAVF characteristics versus treatment

Table 3 presents dAVF type and location versus primary treatment modality of all 112 fistulas, including gender representation of the various dAVFs.

**Table 3:** dAVF characteristics (type and location) versus primary treatment modality in 107 patients with 112 dAVFs

dAVF	Overall		Type I		Type II		Type III		Type IV	
Male *n* (%)	75 (63%)		13 (52%)		21 (72%)		17 (65%)		24 (75%)	
Type of primary treatment	EVT	SUR	EVT	SUR	EVT	SUR	EVT	SUR	EVT	SUR
Primary treatment *n*	72	40	24	1	25	4	13	13	10	22
Primary treatment %	64%	36%	96%	4%	86%	16%	50%	50%	31%	69%
Locations										
Supra-tentorial (male 78%)	13	12	-	-	3	-	6	5	4	7
Superior sagittal sinus	10	6	-	-	3	-	4	1	3	5
Cribriform plate	-	4	-	-	-	-	-	2	-	2
Convexity	2	2	-	-	-	-	1	2	1	-
Sphenoidal	1	-	-	-	-	-	1	-	-	-
Tentorial (male 79%)	4	15	-	-	-	1	-	5	4	9
Tentorial type 1	1	1	-	-	-	1	-	-	1	-
Tentorial type 2	2	7	-	-	-	-	-	4	2	3
Tentorial type 3	-	1	-	-	-	-	-	-	-	1
Tentorial type 4	-	1	-	-	-	-	-	-	-	1
Tentorial type 5	1	4	-	-	-	-	-	1	1	3
Tentorial type 6	-	1	-	-	-	-	-	-	-	1
Transverse-sigmoid-jugular (male 62%)	42	11	17	1	17	2	6	2	2	6
Transverse	4	2	-	1	3	1	1	-	-	-
Transverse-sigmoid angle	19	5	8	-	5	-	4	1	2	4
Sigmoid	15	4	6	-	8	1	1	1	-	2
Jugular bulb	4	-	3	-	1	-	-	-	-	-
Marginal sinus (male 0%)	1	-	-	-	-	-	1	-	-	-
Cavernous sinus (male 50%)	12	2	7	-	5	1	-	1	-	-

Numbers and percentages (%) of females and males are listed for the different dAVF types. Male percentage (%) is listed for the main dAVF locations.

*EVT*, endovascular treatment; *SUR*, surgery.

Primary surgery led to a higher frequency of dAVF occlusion than EVT (90 versus 60%, *p* = 0.003, Table 2). Whereas the residual 29 dAVFs after primary EVT were of all types (type I (*n* = 6), type II (*n* = 13), type III (*n* = 5), type IV (*n* = 5)), the four dAVF residuals after primary surgery were only of type I (*n* = 1) and type II (*n* = 3). Twenty-three of the 33 residual dAVFs had secondary treatment, 20 with secondary surgery and three with secondary EVT (see Table 2). The 107 patients hence underwent totally 157 treatment procedures, 96 endovascular and 61 surgical. Whereas 75 (70%) patients had just one procedure, 23 (21%) underwent two (three patients had treatment of two separate fistulas) and nine had 3–8 procedures. The patient with eight procedures underwent treatment of three separate type II dAVFs.

The endovascular procedures included 73 transarterial-, 18 transvenous-, and five combined transarterial/transvenous procedures. The most frequent arteries used were the middle meningeal artery (*n* = 51), the occipital artery (*n* = 20), and the ascending pharyngeal artery (*n* = 6). Transarterially, liquid embolic materials (Squid® *n* = 40, Onyx® *n* = 25, PHIL® *n* = 5, N-butyl cyanacrylate *n* = 5) and endovascular coils (*n* = 8) were injected/inserted. The transvenous approaches were through the transverse-sigmoid sinus (*n* = 13), the inferior petrous sinus (*n* = 5), and the ophthalmic vein (*n* = 5); coils were deposited in all procedures. Nine of the transvenous procedures were treatment of cavernous sinus dAVFs and included endovascular reopening of a thrombosed inferior petrous sinus (*n* = 3) and surgical exposure for direct puncture of the superior ophthalmic vein (*n* = 3).

The surgical procedures included dAVF leptomeningeal draining vein disconnection (*n* = 47), dAVF excision with resection of the sinus (*n* = 6), dAVF excision only (*n* = 2), and distal extracranial feeding artery disconnection (*n* = 6).

Including primary and secondary treatment as well as the six dAVFs that occluded spontaneously late (> 3 months) after treatment, we obtained a final angiographic obliteration in 104/112 (93%) dAVFs (see Table 2), including 84/85 (99%) dAVFs with leptomeningeal venous drainage (types II/III/IV) that had follow-up angiography—the last dAVF (type II) was converted into a type I fistula with minor symptoms.

Among the six patients with residual dAVF flow after primary EVT but with no angiographic follow-up, two out of four type I dAVF patients became asymptomatic whereas the other two had minor residual symptoms (pulsatile tinnitus). Both patients with type II dAVFs became asymptomatic within weeks after the EVT.

### Presenting and residual symptoms in non-hemorrhagic patients

Presenting and residual symptoms of the 75 non-hemorrhagic patients are listed in Table 4. Headache was a common presenting symptom of dAVFs in all locations whereas pulsatile tinnitus and ocular symptoms were mostly linked to dAVFs located close to the middle ear and the cavernous sinus: in 39/45 and 12/14 cases, respectively. At a median of 10 months (range 2–106 months) after treatment, the symptom intensity was reduced in the 2/45 (4%) patients that still had some pulsatile tinnitus, the 1/14 (7%) patient with some residual ocular symptoms, and in the 4/12 (33%) patients that had some residual neurological symptoms. Residual headache was significantly more common than residuals of other symptoms (*p* < 0.001, Table 4). In the 18 patients with residual headache, intensity had decreased in nine, was unaltered in eight, and increased in one patient as compared to pre-treatment level. Residual headache was present in 6/12 (50%) of those that presented with headache only and in 12/30 (40%) of those that also had other presenting symptoms.

**Table 4:** Presenting and residual symptoms in 75 patients with non-hemorrhagic symptoms in relation to dAVF location

	*n*	Supratentorial	Tentorial	Transverse-sigmoid-jugular	Marginal sinus	Cavernous
*n*		12	8	40	1	14
**Pulsatile tinnitus**						
Presenting	45	5	5	31	-	4
Residual	2	-	-	2	-	-
**Ocular**						
Presenting	14	-	-	2	-	12
Residual	1	-	-	-	-	1
**Neurological**						
Presenting	12	1	1	6	-	4
Residual	4	1	1	1	-	1
**Headache**						
Presenting	42	11	6	15	1	9
Residual	18	5	1	7	1	4

There was some residual dAVF flow in both patients with residual pulsatile tinnitus. Whereas all 14 patients with ocular symptoms had dAVF venous flow into orbital vein(s) prior to treatment, 13 became asymptomatic upon complete closure of their dAVF. In contrast, there was still some dAVF flow into the orbital vein in the patient with some residual ocular symptoms. There was no residual flow in the four patients with residual neurological symptoms or in the 18 patients with residual headache.

### Hemorrhagic patients

The 23 hemorrhagic patients (17, 74% males) presented with one out of two distinct separate symptoms: acute headache (*n* = 19, 83%) or acute neurological symptoms without headache (*n* = 4, 17%). The hemorrhagic dAVFs were all type III (2/23, 9%) or type IV (21/23, 91%); moreover, the DSA of 5/23 (22%) patients had a congestive pseudophlebitic venous appearance (see Fig. 3). There was a close relationship between the presenting symptom and the angiographic findings: In 17/19 (89%) patients with acute headache, the DSA showed a venous aneurysm with a bleb linked to the hemorrhage whereas in 4/4 (100%) patients presenting with acute neurological symptoms without headache, the DSAs had a congestive pseudophlebitic venous appearance. In contrast, this venous appearance was present in only 1/19 (5%) patients presenting with acute headache.

Whereas 20/23 (87%) patients were treated median 18 h (range 3 h–10 days) after ictus, the other three were managed two, seven, and 8 months after the hemorrhage. Type IV dAVFs presented significantly more often with hemorrhage than type III (21/32; 66 versus 2/26; 8%, *p* < 0.001).

There were seven (35%) early rebleeds at median 7.5 h (range 3–96 h) among the 20 patients that were treated in the acute phase. Four of the twenty patients needed emergency haematoma evacuation. Among the other 16 patients, surgery (*n* = 12) was performed significantly faster after arrival than EVT (*n* = 4; median 13.0 versus 35.5 h, *p* < 0.01, Mann–Whitney test). Moreover, 2/4 (50%) of the primary EVT cases needed secondary surgery for complete dAVF closure.

Only 2/23 (9%) patients had symptoms prior to the hemorrhage: one suffered neck pain the last 6 months whereas the other developed change in personality, increasing dementia and gait disturbances over weeks and thereafter a decline in level of consciousness the last 48 h before ictus. Both patients became asymptomatic and recovered fully after treatment.

### Procedural complications

There were 29 (18%) procedural complications in 26 patients: 15 (16%) from EVT and 14 (23%) from surgery (Table 5). The five permanent neurological complications were all cranial nerve (CN) deficits, two complete (CN VII and XII) and three partial (CN VI, VIII, and X). All three deficits after EVT (CN VI, X, and XII) occurred after transarterial embolization using Onyx®. The approaches in the CN X and XII cases were through the ascending pharyngeal artery (dAVFs of the hypoglossal canal/jugular bulb) and in the CN VI case through the middle meningeal—and occipital artery (dAVF along the superior sagittal sinus). In no case did the DSA images, also in retrospect, reveal unwanted catheter position during procedure or excessive reflux or untoward migration of Onyx. Also, follow-up CT and MRI in the patient with CN VI deficit showed no foreign body or ischemia in the brain/brainstem/orbits or along the CN VI origin/route. Both deficits after surgery (CN VII and VIII) occurred in settings with massive bleeding/extensive drilling of the petrous bone combined with dAVF excision and resection of the superior petrous sinus/sigmoid sinus.

**Table 5:** Treatment complications to 96 endovascular and 61 surgical procedures in 107 patients with 112 dAVFs

Total	Endovascular	Surgical
Number of procedures	96	61
Paresis	-	1 (transient)
Cranial nerve dysfunction	4 (1 transient)	2
Seizure	-	2
Postoperative hematoma	3 (1 operated)	2 (1 operated)
Aggravation of symptoms	1 (transient)	1 (transient)
Increase in dAVF type	2 (2 transient)	-
Cranial muscle pain	2	1
Skin dysesthesia	1	2
Corneal erosion	-	1
Embolic material migration	1	
Arterial dissection	1	
Aseptic bone flap necrosis		1
Wound infection		1
**Number of complications**	**15 (16%)**	**14 (23%)**

## Discussion

This study confirmed our hypothesis that our selection of treatment modality (EVT/surgery) depended heavily on the dAVF characteristics and clinical setting. Combining the two modalities gave a high (93%) final angiographic obliteration rate, including all type III/IV fistulas. Outcome was favorable, with 52/77 (68%) patients becoming asymptomatic and 5% permanent cranial nerve deficits.

### Classification of cranial dAVFs

Presently, we used the original dAVF classification of Djindjian, which recently experienced a renaissance [17, 18], probably because it is simple, but still provides the most essential distinctions between dAVF subtypes. The more widely known classifications of Borden et al. and Cognard et al. are both based on the Djindjian system (see Table 1). One may notice that J-J Merland, the senior author of the work by Cognard et al. [10], was also a co-author of the work by Djindjian et al. [13].

The Djindjian classification was able to identify a dAVF subgroup more prone to hemorrhage (type IV) in our material, whereas the hemorrhagic cases would all have been Borden 3 cases without further distinction. A classification according to Cognard would have mirrored that of Djindjian as dAVF types III and IV are identical in the two systems. In contrast, whereas the Djindjan system categorized our 112 dAVFs into just four groups, the Cognard system would have produced seven groups. This further subdividing of groups would not have facilitated or altered our patient advices. Altered patient advice could possibly have occurred in some of the nine patients with incidental dAVFs that we treated if we had applied the concepts by Zipfel et al. [27, 34]. When considering treatment of a dAVF with leptomeningeal drainage, they include the natural history of the specific dAVF type and the case history of that particular patient. This is because a previous history of hemorrhage or non-hemorrhagic neurological symptom (NHNS) from the dAVF grossly increases the risk of future rebleed/NHNS, as compared to patients with benign symptoms/asymptomatic patients [16, 30, 32]. The present experience of 21/23 (91%) of the hemorrhagic cases being asymptomatic prior to ictus, however, suggests elective treatment of asymptomatic type II–IV dAVFs unless patient frailty/medical contraindications exist.

The DES classification system added an extra dimension (congestive pseudophlebitic venous appearance) in our hemorrhagic patients that none of the other systems delineated. This identification and its link to clinical symptoms in hemorrhagic cases are discussed below.

### Patients and dAVF characteristics

The age and male preponderance of our patients correspond with findings of others [2, 8, 18, 26, 29]. Our most frequent dAVF location, along the transverse-sigmoid sinus, also concurs with previous findings [2, 12, 16, 18, 21–23]. The presently high portion of type III/IV dAVFs (52%) probably reflects the combination of a high fraction of hemorrhagic cases (21%) and a low fraction (14%) of patients with type I dAVF and intolerable symptoms.

### Endovascular versus surgical treatment

In accordance with international trends, we used EVT as primary treatment in the majority of dAVFs, including 79% of non-hemorrhagic patients and 91% of types I/II. With treatment of some type I dAVFs being terminated prior to complete occlusion as intolerable symptoms had ceased, our primary EVT angiographic obliteration rate of 60% was lower than that obtained by Gross et al. (76%) [18], Baltsavias and Valavanis (66%) [2], Signorelli et al. (65%) [29], and Guedin/Rodesch (64%) [19], but higher than that of Kirsch et al. (54%) [22] and Piippo (50%) et al. [26]. Our results with primary surgery, perfect (100%) obliteration of type III/IV dAVFs and very poor rates in types I/II, underline both the suitability of surgery in treatment of types III/IV and inadequacy of this modality in treating type I/II dAVFs unless the goal is to convert a type II into type I fistula [33] or merely symptomatic treatment (i.e., pulsatile tinnitus) of a type I dAVF. The present 88% obliteration rate of primary/secondary treatment is similar to the results from combined treatment during the years 2000–2006 of Piippo et al. (81%) [26], but higher than that of Signorelli et al. (27/39, 69%) [29]. Also, the presently spontaneous occlusion of six partially treated dAVFs compares well with that of Gross et al. [17].

Due to its less invasive nature, EVT is a more attractive dAVF treatment modality than surgery. In situations of no procedure-related complications, EVT is very gentle with the brain and leaves no wound so that the patient can usually go home the day after elective treatment and be fit to return to work within days. In contrast, even after successful treatment, a surgically treated patient needs weeks to fully recover. From this, and also based on our dAVF obliteration rates, we recommend EVT as the primary treatment modality in all type I and type II dAVFs. Furthermore, we recommend that EVT should be tried as the first treatment option in non-hemorrhagic type III/IV dAVFs, given appropriate arterial feeder access. Advances during the later years with regard to technical equipment and liquid embolic materials now allow far distal arterial catheter positioning when injecting better controllable embolic agents. This has increased both the proportion of dAVFs being accessible to primary transarterial EVT and the frequency of complete dAVF closures by endovascular means.

Stereotactic radiosurgery (SRS) can be an alternative/adjuvant to elective EVT/surgery of cranial dAVFs, especially of type 1 [9]. This treatment is gentle to the patient, carries low complication risk, and offers favorable obliteration rates over time. It can hence be a treatment of choice in elderly/frail patients. The disadvantage is delayed dAVF obliteration, with a typical latency period of 1–3 years.

Due to the high frequency of early rebleeds among our 20 acute hemorrhagic patients, we recommend that the swiftness of treatment in a hemorrhaging dAVF should be similar to that of aneurysmal subarachnoid hemorrhage. This is independent of whether the symptom at ictus/cause of bleed is acute headache/dAVF venous aneurysm or acute neurological symptoms without headache/bleed from a small parenchymal vein, as we have experienced that both types rebleed early. The longer time span from arrival to primary EVT, together with the lower occlusion success (59%) in EVT, have further made us change our management principles of patients presenting with acute hemorrhage from dAVF; surgery is now our treatment of choice unless directly contraindicated. This contrasts Baltsavias et al. who use primary surgery only in cases of emergency hematoma evacuation [5].

### dAVF characteristics and clinical symptoms

Others have previously described the relationships between dAVF characteristics (lesion location and venous drainage) and neurological symptoms, ocular symptoms, intracranial hemorrhage, and pulsatile tinnitus [3, 4, 12, 22, 23, 25, 28, 29]. When dAVFs recruit cerebral/orbital veins for drainage, increased pressure develops in those compartments and may cause cerebral venous ischemia/neurological symptoms and ocular symptoms. A decompensation of the cerebral venous system may further result in intracranial hemorrhage. In contrast, pulsatile tinnitus is not connected to an increase in pressure but rather to the anatomical intimacy of the dAVF flow to the middle ear. About 85% of our patients that presented with pulsatile tinnitus had dAVFs located in close approximation to the middle ear and the tinnitus persisted when there was residual flow but resolved upon complete dAVF obliteration. Likewise, all our 14 patients who presented with ocular symptoms had dAVF orbital venous drainage. Their symptoms resolved upon complete dAVF closure but persisted if there was some residual dAVF flow. Furthermore, our 12 patients that presented with neurological symptoms or hemorrhage had dAVFs with leptomeningeal venous drainage except for one patient presenting with a partial CN XII deficit. Some residual neurological symptoms in spite of complete dAVF occlusion could possibly indicate a situation of a certain non-reversible neurological damage at the point of time when the dAVFs were treated. The relationship between dAVF and headache seems to be less direct as 43% of our headache patients had residual headache in spite of complete dAVF obliteration.

### Procedural complications

Our frequency of treatment complications was higher than that observed in several other studies [2, 18, 19, 21, 22, 29, 32, 33], comparable to those of Piippo et al. [26] and Gross and Du [16] but lower than that of Kakarla et al. [20].

There were certain common features among our EVT patients that suffered permanent CN deficits: they all underwent transarterial embolization with the low molecular liquid embolic agent Onyx®. All of them were further treated early in the series (November 2012–January 2013) with the available technical equipment at that time and prior to start in our use of the newer generation of liquid embolic agents (Squid® (2014) and PHIL® (2015). Upon closing the dural arterial supply to a region with Onyx®, compounds of this agent may migrate into the vasa nervorum of cranial nerve(s) and cause CN ischemia [14]. If so, one could anticipate the CN deficit to be present early after treatment, which presently was the case. For opacity, Onyx is mixed with tantalum power. However, the rapidly decreasing opacity, as a result of the tantalum powder, may compromise visual control during EVT, which is much more prominent with Onyx than Squid [24]. Lack of full visualization might thus have been contributing to the CN X and XII complications; however, the cause of the CN VI deficit remains unclear. We have had no CN deficits as complication to endovascular dAVF treatment after January 2013.

There were also certain common features in the patients that suffered permanent CN deficit after surgery: both underwent extensive drilling of the petrous bone for sinus exposure/due to massive bleeding and both had the dAVF excised together with resection of a sinus that was located close to the cranial nerve of interest. One may speculate on whether it was the heat produced by the extensive drilling or the coagulation/cutting of dura close to the cranial nerve that led to the deficit. In retrospect, we could have treated both surgical cases differently. The patient with an acute hemorrhaging type IV dAVF that had already undergone unsuccessful primary EVT could have been managed by just clipping of the dAVF draining vein. The other patient suffering from headache and pulsatile tinnitus from a monstrous type II dAVF located along the sigmoid sinus that had already undergone three arterial treatment procedures could have been managed through transvenous endovascular closure of the fistula/sigmoid sinus.

### Limitations

The retrospective nature of this single-center report and the moderate number of patients are limitations of the study. On the other hand, a strength is that we had a low threshold for switching treatment modality when we did not obtain angiographic dAVF obliteration after the first treatment procedure. This reflects our goal of obtaining total obliteration quickly in dAVFs with leptomeningeal venous drainage.

Since our hospital is the national referral center for EVT in neurovascular malformations, there may be a referral bias of dAVF cases deemed suitable for EVT, whereas surgical cases may have been handled locally. If present, this number would be very low, so that the present study includes the vast majority of patients treated for cranial dAVFs in Norway and therefore should adequately reflect features of this disease and treatment outcome.

## Conclusions

From our experience, we recommend EVT as first treatment modality in types I, II, and in non-hemorrhagic type III/IV dAVFs. In contrast, we recommend surgery as first treatment choice in acute hemorrhagic dAVFs, and as secondary choice in type III/IV dAVFs not successfully occluded by EVT. Combining the two modalities, angiographic obliteration can be anticipated in 9/10 patients with dAVF, including all type III/IV dAVFs, at a low treatment risk. Except for headache, relief of non-hemorrhagic symptoms seems to be closely linked to obliteration of the dAVFs.
